# The Role of Endothelial Cell Mitophagy in Age-Related Cardiovascular Diseases

**DOI:** 10.14336/AD.2024.0788

**Published:** 2025-07-26

**Authors:** Quancheng Han, Yiding Yu, Xiujuan Liu, Yonghong Guo, Jingle Shi, Yitao Xue, Yan Li

**Affiliations:** ^1^Shandong University of Traditional Chinese Medicine, Jinan, China.; ^2^Affiliated Hospital of Shandong University of Traditional Chinese Medicine, Jinan, China.

**Keywords:** Endothelial cells, Mitophagy, Aging, Cardiovascular disease (CVD)

## Abstract

Aging is a major risk factor for cardiovascular diseases (CVD), and mitochondrial autophagy impairment is considered a significant physiological change associated with aging. Endothelial cells play a crucial role in maintaining vascular homeostasis and function, participating in various physiological processes such as regulating vascular tone, coagulation, angiogenesis, and inflammatory responses. As aging progresses, mitochondrial autophagy impairment in endothelial cells worsens, leading to the development of numerous cardiovascular diseases. Therefore, regulating mitochondrial autophagy in endothelial cells is vital for preventing and treating age-related cardiovascular diseases. However, there is currently a lack of systematic reviews in this area. To address this gap, we have written this review to provide new research and therapeutic strategies for managing aging and age-related cardiovascular diseases.

## 1. Introduction

Aging is the process of functional decline in organisms as they age [1]. During this process, individuals are more susceptible to diseases and injuries [1-3]. At the cellular level, aging is often accompanied by mitochondrial dysfunction [4], DNA damage [5, 6], reduced cellular metabolism [5], and changes in chromatin structure [7]. Epidemiology shows that age-related cardiovascular diseases remain a leading cause of death worldwide [8, 9]. Aging, as an inevitable risk factor in the progression of cardiovascular diseases, is becoming increasingly prominent with the intensification of global population aging [10]. Therefore, an in-depth study of the mechanisms of interaction between aging and cardiovascular diseases has profound significance for the prevention and treatment of cardiovascular diseases globally.

In recent years, an increasing number of studies have revealed the central role of endothelial cells (ECs) and their functions in cardiovascular health and disease [11-13]. The normal function of ECs is the foundation of cardiovascular health [12, 13]. These cells regulate blood pressure, prevent thrombus formation, and maintain vascular health through various mechanisms, such as modulating vascular tone, preventing platelet aggregation, managing inflammatory responses, and promoting angiogenesis [11, 14-17]. Consequently, endothelial dysfunction is considered a key pathological mechanism in various diseases, such as hypertension, atherosclerosis, and diabetic vascular complications [14, 16]. For instance, ECs dysfunction can lead to reduced nitric oxide (NO) production, affecting the vasodilatory capacity mediated by NO and becoming a critical factor in the pathogenesis of hypertension [14]. Similarly, the action of oxidized low-density lipoprotein (LDL) may damage ECs, leading to the release of chemokines and endothelial adhesion factors, laying the foundation for the development of atherosclerosis and other cardiovascular diseases [18].

Indeed, mitochondrial dysfunction is a core factor leading to the imbalance of ECs function [19]. Given that the content of mitochondria is relatively low in ECs, the stability of mitochondrial function is especially critical for them [19, 20]. Mitochondrial dysfunction manifests in diverse forms, including abnormalities in mitophagy, imbalances in calcium signaling, inadequate energy production, and increased oxidative stress [21]. In particular, abnormal mitophagy is viewed as a key mechanism [22]. Mitophagy, as a selective form of autophagy, is responsible for identifying and eliminating damaged or aged mitochondria, playing an essential role in maintaining the balance of mitochondrial quality and quantity [23]. As the aging process advances, the activity of mitophagy can change [24]. In vascular ECs, aging may decrease the activity of mitophagy, hindering the effective clearance of damaged and aged mitochondria, leading to their accumulation [25-27]. Simultaneously, the biogenesis of new mitochondria also decreases accordingly, ultimately leading to a decline in mitochondrial quality and number, as well as reduced ATP production capacity [28]. These changes can affect the ability of ECs to perform their normal biological functions, thereby leading to endothelial dysfunction and a series of pathological changes, becoming an important pathological mechanism in the development of a variety of cardiovascular diseases.

In this review, we introduce the unique characteristics of mitophagy in ECs and its relationship with aging. The emphasis is on the molecular mechanisms by which EC mitophagy is involved in regulating age-related cardiovascular diseases. This provides a new strategy for the prevention and treatment of related diseases.

## 2. Mitophagy and endothelial function

### 2.1 Structure and function of endothelial cells

#### 2.1.1 Endothelial structure

ECs are a type of squamous cell that constitute the inner wall of blood vessels, covering the internal surfaces of arteries, veins, and capillaries, forming a mechanical barrier between blood and surrounding tissues [15, 29]. These cells have distinct polar features; the side facing the lumen directly contacts the blood components, participating in material exchange and signal transduction [30]. The basal side of ECs interacts closely with the basement membrane through specific cell membrane receptors, such as integrins and focal adhesions [31, 32]. These interactions ensure that ECs are stably attached to the basement membrane [30]. ECs, along with the basement membrane, form the vascular endothelium, essential for maintaining vascular structural integrity and function [33]. Initially, research suggested that ECs were merely a passive barrier isolating blood from tissues [34]. However, as scientific research has progressed, it has been gradually revealed that ECs play a crucial role in regulating vascular tone and blood flow, endocrine functions, inflammatory responses, preventing thrombosis, and angiogenesis, confirming their central regulatory role in the cardiovascular system [35-37].

#### 2.1.2 Endothelial function

**Figure 1.**
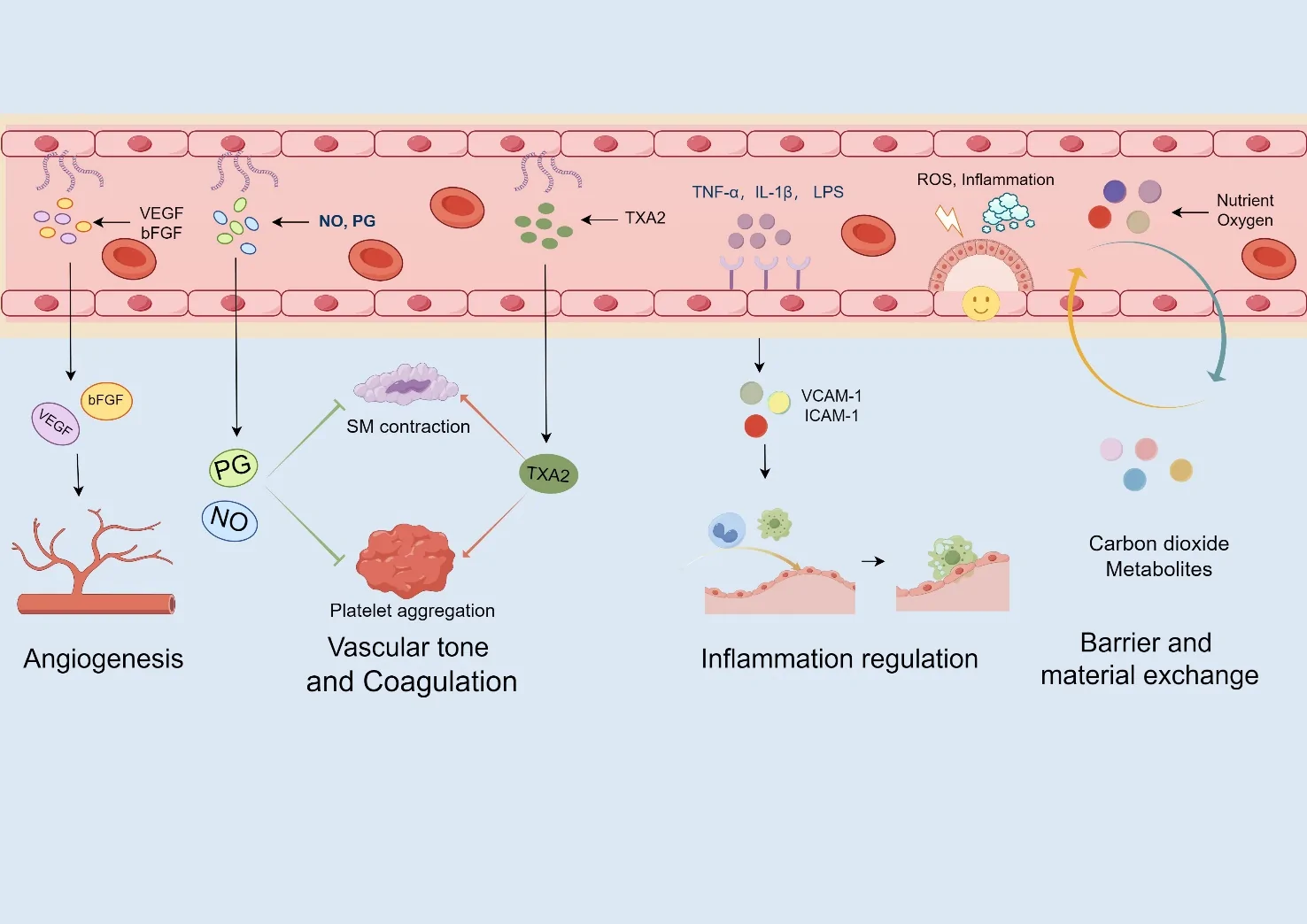
**The functions and regulatory mechanisms of endothelial cells**. The diagram illustrates the key functions of endothelial cells. First, endothelial cells play a crucial role in vascular repair and regeneration, primarily through proliferation, migration, and secretion of factors such as Vascular Endothelial Growth Factor (VEGF) and Basic Fibroblast Growth Factor (bFGF). Second, they are involved in the regulation of vascular dilation and constriction, which is essential for blood pressure regulation, mainly by secreting Nitric Oxide (NO), Prostaglandins (PG), and Thromboxane A2 (TXA2). Third, endothelial cells are critical in blood coagulation and anticoagulation. They synthesize and secrete anticoagulant factors, such as PG and NO, to prevent excessive blood clotting, while also producing pro-coagulant factors, such as TXA2, to address clotting needs during vascular injury. Fourth, endothelial cells contribute to immune regulation by expressing adhesion molecules, such as Intercellular Adhesion Molecule-1 (ICAM-1) and Vascular Cell Adhesion Molecule-1 (VCAM-1), which modulate the adhesion and migration of immune cells. They play a significant role in immune responses, inflammatory processes, and tissue repair. Lastly, endothelial cells are essential for barrier and material exchange functions. They form the endothelial lining of blood vessels, regulating the exchange of substances between blood and surrounding tissues. This barrier function helps maintain vascular permeability, prevents harmful substances and pathogens from entering the bloodstream, and facilitates the exchange of oxygen, nutrients, and metabolic waste products.

Endothelial cells play a multitude of critical biological functions in the human body and are key factors in maintaining the overall health of the vascular system. The primary functions of endothelial cells include regulation of vascular tone and blood flow, blood coagulation and anticoagulation, participation in the modulation of inflammatory responses, angiogenesis and repair, endocrine functions, acting as a barrier in the blood vessel walls, and facilitating substance exchanges. These functions illustrate the multifaceted roles of endothelial cells in regulating vascular health and responding to physiological and pathological processes. The functions and regulatory mechanisms of endothelial cells are shown in Figure 1.

The diagram illustrates the key functions of endothelial cells. First, endothelial cells play a crucial role in vascular repair and regeneration, primarily through proliferation, migration, and secretion of factors such as Vascular Endothelial Growth Factor (VEGF) and Basic Fibroblast Growth Factor (bFGF). Second, they are involved in the regulation of vascular dilation and constriction, which is essential for blood pressure regulation, mainly by secreting Nitric Oxide (NO), Prostaglandins (PG), and Thromboxane A2 (TXA2). Third, endothelial cells are critical in blood coagulation and anticoagulation. They synthesize and secrete anticoagulant factors, such as PG and NO, to prevent excessive blood clotting, while also producing pro-coagulant factors, such as TXA2, to address clotting needs during vascular injury. Fourth, endothelial cells contribute to immune regulation by expressing adhesion molecules, such as Intercellular Adhesion Molecule-1 (ICAM-1) and Vascular Cell Adhesion Molecule-1 (VCAM-1), which modulate the adhesion and migration of immune cells. They play a significant role in immune responses, inflammatory processes, and tissue repair. Lastly, endothelial cells are essential for barrier and material exchange functions. They form the endothelial lining of blood vessels, regulating the exchange of substances between blood and surrounding tissues. This barrier function helps maintain vascular permeability, prevents harmful substances and pathogens from entering the bloodstream, and facilitates the exchange of oxygen, nutrients, and metabolic waste products.

Among the various functions of endothelial cells, constructing the vascular barrier is a fundamental and core capability [29]. Endothelial cells form a barrier through tight junctions among them, which not only facilitate the exchange of substances between blood and tissues but also protect the blood vessels from damage, reflecting the most fundamental function of endothelial cells [38]. By regulating intercellular junctions and contractile proteins, endothelial cells can control the permeability of blood vessels, thereby managing the size of the gaps between cells [39]. This mechanism is beneficial for the regulation of the transport of fluids and large molecular substances to maintain the balance of fluids and materials inside and outside the blood vessels. In pathological states, the integrity of endothelial cells may be compromised. For example, receptors on the surface of endothelial cells, such as Toll-like receptors (TLRs) and cytokine receptors, can specifically recognize cytokines including interleukin-1 (IL-1), tumor necrosis factor (TNF), and bacterial toxins such as lipopolysaccharides (LPS), which can then trigger inflammatory responses [37, 40]. Such responses often lead to changes in endothelial permeability, leakage of white blood cells and large plasma molecules, and damage to the endothelial barrier [29, 37]. In fact, not just inflammation [41, 42], but also factors such as oxidative stress [43], an active RAAS system [44, 45], and uric acid [46, 47] can also cause damage to the endothelium. This damage not only directly affects endothelial cells, leading to endothelial dysfunction, but also, after the endothelium is damaged, causes the scavenger receptors of macrophages to take up toxic oxidized low-density lipoproteins from the blood, accelerate their transformation into foam cells, and ultimately contribute to the formation of atherosclerotic plaques, setting the stage for atherosclerosis [48].

The secretion of hormones is an important way in which endothelial cells perform their functions [49]. These cells release hormones such as nitric oxide (NO), prostacyclins (PG), thromboxane A2 (TXA2), and vascular endothelial growth factor (VEGF), which play a role in regulating cardiovascular health and disease [49, 50]. NO and PG can interact with a variety of receptor cells, thereby increasing the levels of second messengers such as cyclic guanosine monophosphate (cGMP) and cyclic adenosine monophosphate (cAMP) [51]. When acting on platelets, this mechanism can prevent platelet activation, adhesion, and aggregation [51]. In vascular smooth muscle cells, the effects of NO and PG cause smooth muscle relaxation that is cGMP or cAMP-dependent, leading to vasodilation [52, 53]. It is noteworthy that NO and PG exhibit a synergistic effect in platelets, while they have an additive effect in the vascular system [52, 54]. In addition, nitric oxide plays a key signaling role in angiogenesis activated by VEGF [55]. In contrast, TXA2 also influences platelets and vascular smooth muscle cells, but its mechanism is quite different. TXA2 promotes platelet activation and vascular contraction by activating phospholipase C (PLC) and Rho Guanine Nucleotide Exchange Factors (RhoGEF), which stand in stark contrast to the vasodilatory and platelet aggregation inhibitory actions of NO and PG [56]. In summary, endothelial cells play a key role in promoting and inhibiting coagulation and regulating vascular tone through their hormone secretion functions.

Angiogenesis is a complex, multi-stage process that plays a critical role in pathological processes such as wound healing, ischemia/reperfusion injury, and cardiovascular diseases [57]. It includes activation, vascular sprouting and growth, as well as maturation and stabilization of the blood vessels [58-60]. Endothelial cells are indispensable for maintaining vascular homeostasis, remodeling, and angiogenesis [61]. They can secrete a variety of key factors that promote angiogenesis, including vascular endothelial growth factor (VEGF) and basic fibroblast growth factor (bFGF) [62]. These factors trigger the migration and proliferation of peripheral precursor cells, leading to the formation of new blood vessels. During the initial stage of angiogenesis, endothelial cells promote the formation of new blood vessels through migration and proliferation [63]. They are capable of splitting off from existing vessels, moving to new locations, and forming tubular structures to lay down the pathways for blood flow, establishing the basic framework of the vessels [64]. Moreover, endothelial cells are responsible for the stability and functional maintenance of new blood vessels, ensuring the normal operation of the vascular system by regulating vascular permeability and secreting factors that affect vascular tension [65].

The function of endothelial cells is influenced by various factors, including mitochondrial dysfunction, oxidative stress, hemodynamic shear stress, and inflammation [66-68]. Dysfunction of endothelial cells is closely linked to the development of a multitude of diseases, such as atherosclerosis, pulmonary arterial hypertension, and vascular complications of diabetes [69, 70]. Therefore, maintaining the normal function of endothelial cells is of great importance for promoting vascular health and preventing related diseases. The regulatory mechanism of endothelial function is shown in Figure 1.

### 2.2 Mitophagy

#### 2.2.1 Molecular mechanisms of mitophagy

**Figure 2.**
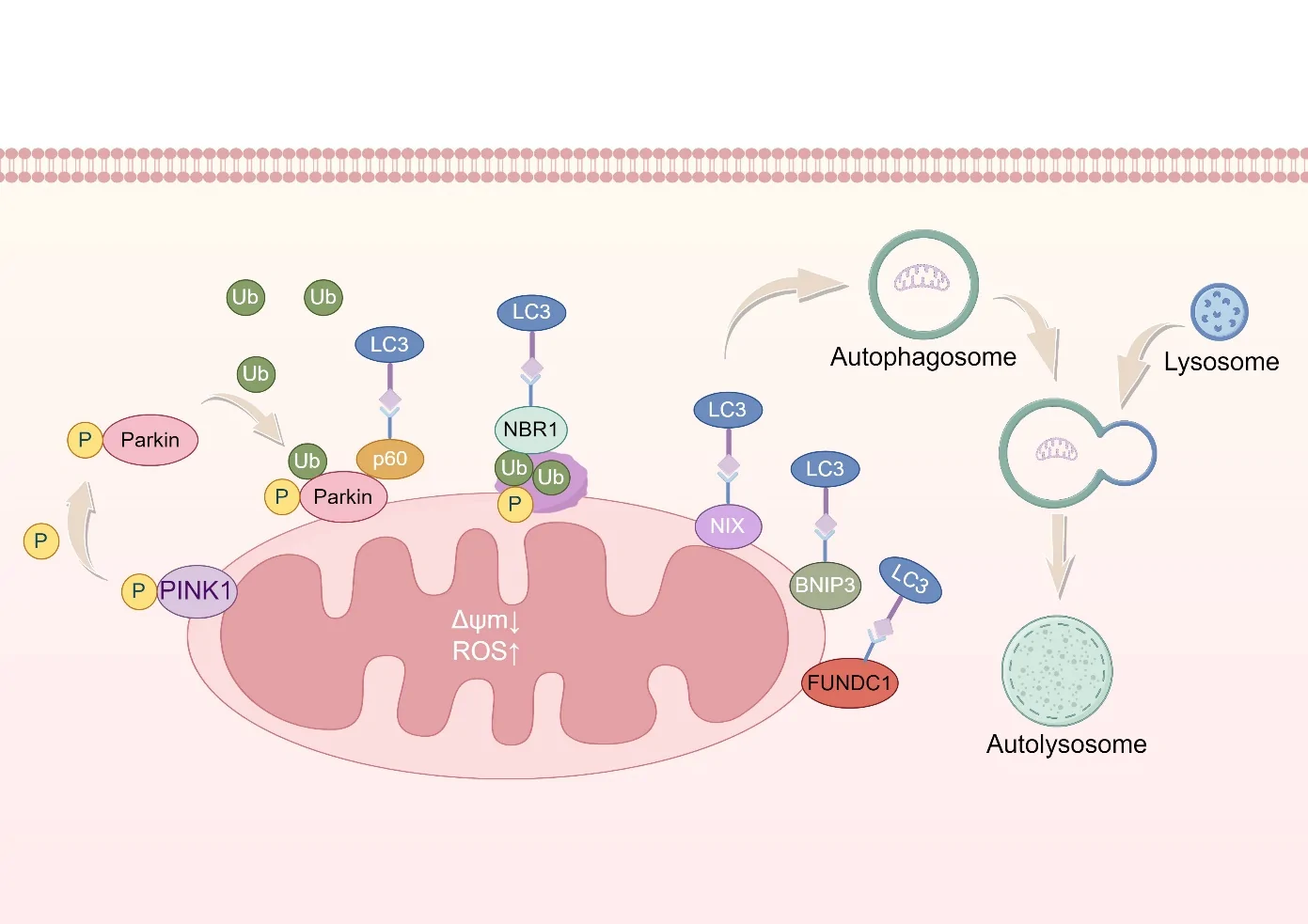
**The regulatory mechanism of mitophagy**. The diagram illustrates the major pathways of mitophagy. PTEN-induced kinase 1 (PINK1) accumulates on the outer membrane of damaged mitochondria and recruits Parkin (an E3 ubiquitin ligase). Parkin tags the damaged mitochondrial proteins with ubiquitin, marking them for autophagic degradation. The autophagosome then fuses with the lysosome to complete the mitochondrial degradation process. NIX (BNIP3-like) is a mitochondrial outer membrane protein that becomes upregulated during mitophagy. NIX enhances the removal of damaged mitochondria by binding with autophagy-related proteins to promote the formation of mitophagosomes. FUN14 domain containing 1 (FUNDC1) and BNIP3 pathways are similar to NIX in their role in mitophagy. Neighbor of BRCA1 gene 1 (NBR1) is an autophagy adaptor protein that binds to ubiquitinated mitochondria and promotes the encapsulation and removal of damaged mitochondria by interacting with LC3 (microtubule-associated protein 1A/1B-light chain 3).

Mitophagy is a selective autophagy process, whose primary function is to remove damaged or dysfunctional mitochondria to ensure the maintenance of mitochondrial quality and quantity within the cell [71]. Following mitochondrial damage leading to a decrease in membrane potential, specific mitochondrial membrane receptors or ubiquitin molecules covalently bound to mitochondrial surface proteins become activated [72]. This promotes the formation of autophagic vesicles that envelop the damaged mitochondria [72]. Subsequently, the autophagosomes surrounding the mitochondria fuse with lysosomes, resulting in the degradation of the mitochondria by lysosomal enzymes, with the breakdown products then being recycled by the cell [73, 74]. The regulatory mechanisms of mitophagy involve various molecules and signaling pathways, with the most significant being the ubiquitin-dependent pathway and the ubiquitin-independent pathway. The regulatory mechanism of mitophagy is shown in Figure 2.

#### 2.2.2 Ubiquitination dependent pathway mitophagy

The ubiquitin-dependent pathway of mitophagy is primarily regulated by the PINK1/Parkin protein system. PTEN-induced putative kinase 1 (PINK1) is a serine/threonine kinase located on the outer mitochondrial membrane, which plays an essential role in response to mitochondrial damage [75]. Parkin RBR E3 ubiquitin protein ligase (Parkin) is an E3 ubiquitin ligase that can attach ubiquitin molecules to target proteins, thus completing the ubiquitination process [76]. Under physiological conditions, the expression and protein activity of PINK1 are relatively low, mainly because its N-terminal mitochondrial targeting signal is recognized and bound by the translocase of the outer membrane (TOM), which guides PINK1 into the mitochondria [77-79]. After crossing the outer mitochondrial membrane, PINK1 further enters the mitochondrial matrix with the help of the translocase of the inner membrane (TIM) [80]. Finally, the targeting signal of PINK1 is cleaved by the Presenilins-Associated Rhomboid-Like protein (PARL), a protease located at the mitochondrial inner membrane, releasing PINK1 fragments back into the cytosol for degradation [71]. Certain pathological changes, such as oxidative stress, mitochondrial genetic mutations, and energy metabolism disorders, can damage mitochondria, causing a decrease in mitochondrial membrane potential [72, 81]. At this point, TIM-mediated translocation of PINK1 is impaired, but its association with TOM is retained, ultimately leading to the accumulation of PINK1 on the outer mitochondrial membrane [82]. The PINK1 protein has autophosphorylation ability and can phosphorylate various proteins, including Parkin [83, 84]. Phosphorylation by PINK1 promotes the translocation of Parkin from the cytosol to the outer mitochondrial membrane (OMM) [85]. Recent research suggests that PINK1 amplifies the mitochondrial recruitment of Parkin by phosphorylating ubiquitin (Ub) and ubiquitin complexes on the OMM [86-88]. This process fully activates Parkin, which then mediates the ubiquitination of proteins on the surface of damaged mitochondria [89]. The ubiquitinated mitochondrial proteins are recognized and bound by ubiquitin-binding autophagy receptors (such as p62/SQSTM1 and NBR1), which also contain LC3-interacting regions, thereby enabling interaction with the autophagosomal membrane protein microtubule-associated protein 1A/1B-light chain 3 (LC3), recruiting the damaged mitochondria to the autophagosome [90, 91]. The autophagic vesicle then fuses with lysosomes, which contain degradative enzymes [73]. The active enzymes within the lysosome break down the contents of the autophagic vesicles into basic biological molecules, such as amino acids, for recycling and reuse [74].

#### 2.2.3 The non-ubiquitylation dependent pathway mitophagy

Some proteins with an LC3-interacting region (LIR), such as Bcl-2/adenovirus E1B 19 kDa interacting protein 3 (BNIP3), Bcl-2/adenovirus E1B 19 kDa interacting protein 3-like (BNIP3L, also known as NIX), and FUN14 Domain Containing 1 (FUNDC1), can directly recruit autophagosomes containing LC3 without the need for ubiquitination, thereby triggering mitophagy [92, 93]. This process is different from the PINK1/Parkin-mediated mitophagy initiation mechanism. These non-ubiquitin-dependent pathways are mainly activated under specific conditions, such as hypoxia or nutrient deprivation [94, 95]. This mechanism ensures that cells can flexibly respond according to different stress conditions and states, efficiently identifying and clearing damaged mitochondria to maintain cellular health and prevent cell death caused by impaired mitochondria.

BNIP3, a member of the BCL-2 protein family, is primarily involved in regulating apoptosis and plays a significant role in mitophagy [93]. It has a typical LIR at its N-terminus, which can directly interact with LC3, thereby initiating the mitophagy process [93]. Under normal conditions, BNIP3 generally exists within cells in a monomeric form, in an inactive or partially active state [96]. However, under conditions such as hypoxia, hypoxia-inducible factor-1 alpha (HIF-1α) enters the nucleus and upregulates the expression of BNIP3 [97]. This leads to the transmembrane domain-mediated oligomerization of adjacent BNIP3 molecules [93, 96]. In mitophagy, oligomerization of BNIP3 promotes its interaction with the LC3 protein, thus recruiting autophagosomes and causing mitophagy [93]. NIX, belonging to the same family as BNIP3, is also a regulator of mitophagy and has overlapping or complementary functions with BNIP3 [98]. NIX's regulatory mechanisms are similar to those of BNIP3, primarily involving interactions with LC3 to regulate mitophagy [96]. In addition to transcriptional regulation by HIF-1α, BNIP3 and NIX also participate in the Parkin-mediated ubiquitin-dependent pathway, thereby promoting mitophagy [99].

FUNDC1 is an OMM protein, with an N-terminal LIR exposed to the cytosol, serving as a receptor for LC3 in mitophagy [92]. The activity and function of FUNDC1 depend on the phosphorylation and dephosphorylation of its LIR [100]. Under normal circumstances, FUNDC1's phosphorylation levels are high, inhibiting its interaction with the autophagy-related protein LC3 and thus controlling mitophagic activity [94]. Under hypoxic conditions, some kinases, such as CK2 and Src, become inactivated, leading to the dephosphorylation of FUNDC1 at the phosphorylation sites Ser13 and Tyr18 [92, 94, 101]. At the same time, hypoxia induces the recruitment of AMPK to the mitochondria, which phosphorylates FUNDC1 at Ser17 by Unc-51 Like Autophagy Activating Kinase 1 (ULK1), exerting a positive regulatory effect on mitophagy [102]. In addition, phosphoglycerate mutase family member 5 (PGAM5) can also be activated under hypoxic conditions, dephosphorylating the Ser13 site [94]. All the above processes promote the interaction between FUNDC1 and LC3, leading to mitophagy.

Apart from the aforementioned pathways, cardiolipin (CL) [103], BCL2-like 13 (BCL2L13) [100], FK506 Binding Protein 8 (FKBP8) [104], Inhibin Beta B [105], Autophagy and Beclin 1 Regulator 1 (AMBRA1) [106], Choline Dehydrogenase (CHDH) [107], and others can also regulate mitophagy through various mechanisms. In summary, the multiple regulatory pathways of mitophagy ensure the accuracy and adaptability of the mitophagy process when cells face various stress factors. This mechanism allows for the timely clearance of damaged mitochondria, preventing cell death due to mitochondrial dysfunction. The mechanism of mitophagy is shown in Figure 2.

### 2.3 Relationship between mitochondrial autophagy and other cell phenotypes

#### 2.3.1 Mitophagy and Inflammation

Mitophagy reduces intracellular oxidative stress and inflammatory responses by removing damaged mitochondria. Damaged mitochondria release pro-inflammatory factors such as mitochondrial reactive oxygen species (mtROS) and mitochondrial DNA (mtDNA), which can activate inflammatory pathways [108]. Mitophagy-related proteins, like PINK1 and Parkin, mark damaged mitochondria for clearance, thereby reducing the release of these pro-inflammatory factors and inhibiting inflammation. Research has shown that dysregulation of mitophagy is associated with various inflammatory diseases, such as atherosclerosis [109]. In cardiovascular diseases, exacerbated inflammatory responses can damage endothelial cells, promoting atherosclerosis and the progression of heart disease. Therefore, maintaining normal mitophagy function is crucial for controlling cardiovascular inflammation.

#### 2.3.2 Mitophagy and Oxidative Stress

Oxidative stress is a significant pathological process in cardiovascular diseases, usually caused by excessive reactive oxygen species (ROS). Mitochondria are the primary source of ROS, and damaged mitochondria release more ROS, thereby exacerbating oxidative stress [110]. Mitophagy reduces ROS production by clearing damaged mitochondria, thereby lowering oxidative stress levels [110]. Studies have found that decreased mitophagy leads to increased oxidative stress, resulting in cardiomyocyte apoptosis and a decline in cardiac function [111]. In cardiovascular diseases such as heart failure and coronary artery disease, mitophagy dysregulation exacerbates oxidative stress, which has been shown to be a critical factor in disease progression [111]. Regulating mitophagy can effectively reduce oxidative stress and improve outcomes in cardiovascular diseases.

#### 2.3.3 Mitophagy and Apoptosis

There is a complex interaction between mitophagy and apoptosis. Under normal circumstances, mitophagy maintains cellular energy metabolism and function by removing damaged mitochondria, thereby inhibiting apoptosis. However, when mitophagy is dysfunctional, damaged mitochondria accumulate and release pro-apoptotic factors such as cytochrome c and apoptosis-inducing factor (AIF), which activate apoptotic pathways [112]. In cardiovascular diseases, cardiomyocyte apoptosis is closely related to the loss of cardiac function. Research indicates that defects in mitophagy can lead to excessive cardiomyocyte apoptosis, worsening heart failure and other cardiovascular disease progressions [112]. Therefore, regulating mitophagy to maintain cardiomyocyte health has potential therapeutic value.

### 2.4 Characteristics of mitophagy in endothelial cells

In addition to endothelial cells, other types of cells in the cardiovascular system also play important physiological roles, such as smooth muscle cells, macrophages, and cardiomyocytes. Mitophagy of endothelial cells has many basic mechanistic similarities with mitophagy of macrophages, smooth muscle cells, and cardiomyocytes, but there are also some important differences. Macrophages are responsible for engulfing and clearing pathogens, dead cells, and other foreign particles in the immune system. Mitophagy in macrophages not only helps remove damaged mitochondria but also affects macrophage polarization and cytokine secretion [113]. Therefore, mitophagy in macrophages is critical for regulating immune function and inflammatory responses. Smooth muscle cells, found in the smooth muscle tissues of blood vessels and other organs, are responsible for maintaining vascular tone and organ contraction. Mitophagy in smooth muscle cells is crucial for maintaining cellular function and adapting to stress [114]. Dysregulation of mitophagy in smooth muscle cells may be associated with diseases such as vascular remodeling and atherosclerosis. Cardiomyocytes have high energy demands, with mitochondria playing a key role in energy supply within these cells [115]. Mitophagy in cardiomyocytes directly impacts cardiac function and the response to heart diseases, particularly concerning heart failure and ischemic heart disease.

Compared with these types of cells, mitochondrial autophagy in endothelial cells has some unique functions. Endothelial cells are in direct contact with the blood environment and are susceptible to damage from oxidative stress and inflammation [116]. Mitophagy can remove damaged mitochondria, reducing oxidative stress and inflammation levels in endothelial cells, thereby protecting them from harm [117]. In addition, mitophagy regulates the metabolism and signaling pathways of endothelial cells, influencing the hormones they secrete and thereby affecting functions such as vascular tension, coagulation, thrombosis, and angiogenesis [118, 119]. Mitophagy also reduces the level of inflammation within cells, thereby minimizing inflammatory damage to endothelial cells [120]. In summary, mitophagy plays a key role in maintaining the functional stability of endothelial cells.

## 3. The impact of mitophagy in vascular endothelial cells on cardiovascular diseases during the aging process.

As the aging process progresses, mitophagy impairment in endothelial cells leads to the ineffective clearance of damaged mitochondria, resulting in the excessive generation of ROS [119, 121, 122]. ROS can damage intracellular proteins, lipids, and DNA, thereby accelerating cellular aging [123]. Additionally, mitochondria are the primary energy suppliers for cells. Impaired mitophagy reduces the number and functionality of mitochondria, decreasing ATP production and affecting cellular energy metabolism [28]. This energy deficiency leads to a decline in endothelial cell function, which promotes the aging process. Furthermore, dysfunctions in key mitophagy pathways (such as the PINK1/Parkin pathway, NIX pathway, and FUNDC1 pathway) affect mitochondrial quality control, leading to mitochondrial stress and endothelial cell aging [24]. Importantly, damaged mitochondria release endogenous danger signals (such as mitochondrial DNA and ATP), which activate intracellular inflammatory response pathways [108]. Excessive release of inflammatory factors exacerbates endothelial cell aging, thereby further deteriorating vascular function.

Aging significantly impairs endothelial cell (EC) function, including reduced vasodilation capacity, increased vascular permeability, and decreased response to inflammation and injury. Additionally, aging endothelial cells produce more pro-inflammatory factors, such as endothelin-1 and cytokines (such as IL-6 and TNF-α), leading to local and systemic chronic inflammation [26]. Aging is also accompanied by increased levels of oxidative stress, with higher ROS production further damaging cellular functions [124]. Aging also affects the repair and regeneration capabilities of endothelial cells, impacting vascular repair and regeneration [125]. These changes constitute the pathological basis for many age-related cardiovascular diseases.

In cardiovascular diseases such as coronary artery disease and stroke, endothelial cell aging directly leads to endothelial barrier damage and increased vascular wall permeability, allowing plasma components (such as cholesterol and low-density lipoprotein) to enter the vascular wall and trigger atherosclerosis [49]. Additionally, due to EC aging, secretory functions are impaired, resulting in reduced NO production, decreased vasodilation capacity, and increased risks of vasoconstriction and hypertension [49]. Aging endothelial cells release pro-inflammatory factors (such as endothelin-1, IL-6, and TNF-α), triggering local inflammatory responses [49]. Inflammatory cells, such as monocytes and macrophages, migrate to the vascular intima, forming atherosclerotic plaques. Endothelial cells upregulate adhesion molecules (such as ICAM-1 and VCAM-1), promoting the attachment and entry of inflammatory cells into the vascular wall, thereby further driving the progression of atherosclerosis [126]. Meanwhile, increased levels of oxidative stress in endothelial cells and ROS production lead to damage to endothelial cells and the vascular wall. These ROS oxidize LDL, forming oxidized LDL, which promotes plaque formation and atherosclerosis [126]. Thus, EC aging is a significant risk factor for various cardiovascular diseases. Moreover, aging is also a risk factor for mitophagy impairment, as aging endothelial cells lead to mitophagy dysfunction, and mitophagy impairment also leads to endothelial cell aging, mutually influencing each other and ultimately causing endothelial dysfunction.

Due to mitophagy impairment, excessive accumulation of mitochondrial ROS results in a series of cellular damages. In a recent clinical trial, the use of mitochondrial antioxidants significantly improved vascular endothelial function in elderly individuals, primarily by reducing oxidized LDL, thereby increasing nitric oxide levels and reducing mitochondrial oxidative stress in endothelial cells [127]. Furthermore, as mitophagy impairment activates inflammatory pathways, another clinical study demonstrated that NLRP3 inflammasome-mediated inflammatory responses promote endothelial cell aging [128]. This fully illustrates the importance of regulating mitophagy to delay endothelial cell aging and treat cardiovascular diseases. Here, we focus on discussing the role of mitophagy in atherosclerosis, stroke, and diabetic vascular injury, as well as the corresponding regulatory measures.

**Table 1:** Progress in endothelial mitophagy.

Molecules/Heal	Model	Modeling method	The impact of mitophagy	Function	References
**Empagliflozin**	CMEC	H/R	AMPKα1, ULK1, and FUNDC1 upregulated	Enhanced mitophagy, protected mitochondrial function, and attenuated microvascular I/R injury in the heart	148
**Urolithin A**	HUVEC	H/R	Sirtuin 3 upregulated	Enhanced mitochondrial autophagy improves mitochondrial quality control and increases cell viability and proliferation to reduce I/ R-induced cardiac microvascular damage	150
**Resveratrol**	HUVEC	Oxidized LDL	HIF1, AMPK, and BNIP3 upregulated	Enhancement of mitophagy reduces hyperlipidemia-associated endothelial damage	151
**NR4A1**	RAEC	Oxidized LDL	Parkin upregulated	Over-enhancement of mitophagy, the deficiency of NR4A1 can protect AECs from ox-LDL induced apoptosis by inhibiting excessive mitophagy	147
**Rcan1-1L**	HUVEC	Hypoxia	Parkin upregulated	Enhanced mitophagy helps cells survive under hypoxic conditions	146
**Urolithin A**	EA.hy926	CuONPs	PINK1, LC3B upregulated	Enhancing mitophagy alleviates pathological endothelial damage caused by exposure to Copper oxide nanoparticles	153
**UCP2**	MEC	High-salt	LC3-II/LC3-I, Parkin up-regulated	Enhancing mitophagy counteracts oxidative stress damage induced by high salt	154
**IDH2**	HUVEC	α-KG	PINK1, Parkin, LC3-II/LC3-I, and p62 upregulated	Enhancing mitophagy resists oxidative stress and delays endothelial cell aging	155
**CRIF1**	HUVEC	CRIF1 knockdown	LC3-II/LC3-I, PINK1, Parkin, and p66Shc up-regulated	CRIF1 deficiency activated mitophagy, exacerbating oxidative stress damage in endothelial cells	161
**YMG**	HUVEC	AngⅡ	Pink1, Mfn2, Parkin upregulated	Enhanced mitophagy regulates pro-inflammatory factors, vasoconstrictor cytokines, and blood lipids, thereby reducing atherosclerosis	1561
**Melatonin**	CMEC	H/R	AMPKα up-regulated, Drp1, PINK1, and Parkin down-regulated	By activating AMPKα to inhibit excessive mitophagy, endothelial cells are protected	162
**Vitamin D**	HUVEC	HG and PM	LC3B, BNIP3, ICAM-1, VCAM-1 expressions are decreased	Inhibiting mitophagy, oxidative stress, and inflammatory responses protects endothelial cells	164
**Microgravity**	HUVEC	mdivi-1, PINK1 knockdown	PINK1 down-regulated, NLRP3 up-regulated	Some error occurred Please try again or contact the support team in our communities	165
**Exercise**	CMEC	H/R	FUNDC1 upregulated	Exercise enhances FUNDC1-mediated mitophagy to protect endothelial cells from ischemia-reperfusion injury	26
**Low shear stress**	RAEC	Parallel plate chamber system	SQSTM1, PINK1, Parkin, and LC3-II down-regulated	Inhibiting mitophagy leads to the promotion of endothelial cell damage	167
**Roger**	bEnd.3 cells	H/R	SIRT3, Pink1, Parkin upregulated	Activation of mitochondrial autophagy after cerebral ischemia-reperfusion promotes angiogenesis	178
**MG**	bEnd.3 cells	HG	Parkin-1, HIF-1α, LC3-II/LC3-I up-regulated	It leads to mitochondrial damage and autophagy of endothelial cells, and promotes endothelial cell damage	183
**tPA**	bEnd.3 cells	H/R	FUNDC1 upregulated	Increased mitochondrial autophagy plays a neuroprotective role	179
**FA**	BMEC	OGD	LC3-II up-regulated	Promoting mitochondrial autophagy mitigated OGD-induced mitochondrial oxidative damage.	174
**PDK1**	MVEC	Hypoxia	PINK1, Parkin upregulated	Excessive activation of mitochondrial autophagy aggravates oxidative stress injury of endothelial cells	182
**Nanocurcumin**	HUVEC	HG and PM	Bnip3 upregulated	Enhance mitochondrial autophagy and inhibit inflammation	160
**Nicorandil**	HUVEC	HG	Pink1, Parkin upregulated	Promote mitochondrial autophagy to inhibit mitochondria-associated iron death	189
**FGF13**	GEC	HG	Parkin upregulated	Inhibit mitochondrial autophagy, promote apoptosis and accelerate endothelial damage	192
**adiponectin**	PMVEC	H/R	SIRT1,PINK1 up-regulated	Activation of mitochondrial autophagy alleviates oxidative stress, inflammation, apoptosis and mitochondrial dysfunction in H/R injury.	190
**BDNF**	BMEC	HG	LC3-II, p62, HIF-1α, BNIP3up-regulated	Enhanced mitochondrial autophagy plays a protective role in BMEC injury under hyperglycemia	182,198
**MSC**	HUVEC	HG	PINK1, Parkin upregulated	Enhancing mitochondrial autophagy improves mitochondrial dysfunction and protects endothelial cells from hyperglycemia-induced damage	197
**Liraglutide**	HUVEC	HG	PINK1, Parkin upregulated	Inhibit mitochondrial autophagy and counteract high glucose induced cell dysfunction	199
**Sal B**	ECs	HG and oxLDL	ROCK1 downregulated	Inhibition of mitochondrial autophagy reduces apoptosis-related proteins and protects endothelial function	192
**Scutellarin**	HUVEC	HG	LC3 II, PINK1, Parkin upregulated	Mitochondrial autophagy is upregulated to protect vascular endothelial cells from damage caused by hyperglycemia	200
**H2S**	RAEC	HG/HP	LC3B, PINK1, Parkin upregulated	Promote mitochondrial autophagy and protect RAEC from apoptosis under high glucose and palmitic acid conditions	196
**L-carnitine**	HCMEC	HG/FFA	PINK1, Parkin upregulated	Enhanced mitochondrial autophagy, thereby reversing mitochondrial dysfunction and cardiac microvascular damage in diabetic cardiomyopathy	191
**GSC**	HAEC	HG/PA	PINK1, Parkin, LC3B-II upregulated	Promote mitochondrial autophagy and prevent HG/ Pa-induced endothelial senescence and mtROS production	195

### 3.1 Coronary heart disease (CHD)

Coronary heart disease (CHD) is a progressive condition characterized by coronary artery injury and is the primary cause of myocardial infarction [129, 130]. The main pathological changes include endothelial dysfunction, lipid accumulation, foam cell proliferation, inflammatory responses, and plaque formation and development [131-135]. Among these, endothelial dysfunction—particularly endothelial barrier damage and the accumulation of adhesion proteins—is considered the core pathological change at the onset of CHD [136-138]. Studies have shown that with aging, the level of mitophagy in endothelial cells of the coronary arteries and aorta is reduced, leading to the accumulation of damaged mitochondria. This manifests as reduced bioavailability of nitric oxide (NO), increased oxidative stress, and enhanced inflammatory responses [26, 123]. Therefore, activating mitophagy in endothelial cells to clear damaged mitochondria has become a key strategy in the treatment of CHD.

### 3.1.1 Endothelial Cell Mitophagy and Oxidative Stress

Oxidative stress is a pathological state caused by an imbalance in the intracellular and extracellular redox systems [139]. It is usually associated with the accumulation of reactive oxygen species (ROS), including superoxide anions, hydroxyl radicals, and hydrogen peroxide [140]. ROS production mainly occurs in the mitochondrial inner membrane's electron transport chain (ETC) [110]. Under normal conditions, ROS generated by mitochondria are reduced by cellular antioxidants such as superoxide dismutase (SOD) and glutathione peroxidase (GPx) [141]. However, when mitochondria are damaged, dysfunction of the electron transport chain leads to electron leakage and hence an increased production of ROS [142]. When damage accumulates to a certain degree beyond the capacity of the redox regulatory ability, oxidative stress occurs, leading to damage to intracellular lipids and proteins [143]. Endothelial cells, being in direct contact with the blood environment, are vulnerable to damage by oxidative stress and inflammation [144, 145]. Components in the blood, such as oxidized low-density lipoprotein and lipopolysaccharide, hypoxia, and ischemia-reperfusion injury, can all cause oxidative stress in endothelial cells, leading to endothelial dysfunction [146-149].

Cardiac ischemia-reperfusion injury activates oxidative stress responses in cardiac microvessels, resulting in an increase in ROS levels, which then disrupts endothelial barrier integrity, leading to disordered secretion of hormones such as NO and ET-1, and also affecting the mitophagy function of endothelial cells [148]. However, Empagliflozin reverses endothelial cell damage and dysfunction by activating mitophagy dependent on the AMPKα1/ULK1/FUNDC1 pathway [1148]. Moreover, the mitophagy inducer Urolithin A (UA) protects mitochondrial function by reducing mitochondrial oxidative stress and stabilizing the mitochondrial membrane potential in endothelial cells [150]. These findings suggest that mitophagy plays a protective role against endothelial cell dysfunction caused by ischemia-reperfusion injury.

Oxidative stress also occurs in endothelial cell damage induced by ox-LDL [151]. Resveratrol upregulates BNIP3 via HIF1 and AMPK, enhancing mitophagy and alleviating endothelial damage caused by oxidative stress [151]. This suggests that at the early stages of atherosclerosis, high levels of low-density lipoprotein may cause damage to endothelial cells, but such damage can be repaired by regulating mitophagy, thus slowing the progression of coronary heart disease. However, the role of mitophagy in ox-LDL-induced endothelial cell damage is still debated. Research by Li et al. observed that the expression of NR4A1 (Nuclear Receptor Subfamily 4 Group A Member 1) in aortic endothelial cells treated with ox-LDL results in the excessive activation of Parkin-mediated mitophagy, leading to a significant reduction in healthy mitochondria [147]. This eventually causes endothelial cell damage due to energy shortage and mitochondrial dysfunction, exacerbating the progression of CHD [147]. This reflects the complexity of mitophagy regulation, where moderate mitophagy helps maintain cell survival, while excessive or insufficient levels may lead to pathological states. Therefore, exploring the balance between insufficient and excessive endothelial cell mitophagy is of great significance for the prevention and treatment of CHD.

In addition to the aforementioned causes of oxidative stress in endothelial cells, other stimuli can also induce oxidative stress in these cells. For example, hypoxia can cause oxidative stress by producing excessive ROS due to the accumulation of damaged mitochondria. Calcineurin phosphatase 1-1L (Rcan1-1L) helps endothelial cells survive under hypoxic conditions by initiating mitophagy [146]. Copper oxide nanoparticles (CuONPs) are a type of metal oxide nanoparticle that can directly trigger oxidative stress and inflammation [152]. The mitophagy activator Urolithin A promotes the clearance of damaged mitochondria induced by mtROS and CuONPs, thereby protecting vascular endothelial cells from damage [150]. In the case of excessive ROS accumulation in endothelial cells caused by high salt exposure, mitochondrial uncoupling protein 2 (UCP2) can counteract the oxidative stress damage induced by high salt through enhancing mitophagy [153]. Isocitrate dehydrogenase 2 (IDH2) is an enzyme located in the mitochondria, involved in the citric acid cycle and closely related to energy production [154]. IDH2 can resist oxidative stress and regulate endothelial aging by increasing the levels of mitophagy markers PINK1, Parkin, and LC3-II/LC3-I in endothelial cells through regulating the accumulation of miR-34b/c [154]. Additionally, studies show that the absence of mitochondrial protein CR6 interacting factor 1 (CRIF1) leads to mitophagy disorder and ROS accumulation, but the downregulation of p66shc can restore mitochondrial dysfunction, while increasing the levels of LC3-II/I, PINK1, and Parkin, thus reducing CRIF1 defect-induced mitophagy [155]. In summary, oxidative stress in endothelial cells can be triggered by a variety of factors, leading to endothelial dysfunction. Mitophagy can alleviate the damage caused by oxidative stress, thus protecting the endothelium. Therefore, regulating mitophagy can serve as an important prevention and treatment strategy for early endothelial damage in CHD.

### 3.1.2 Endothelial Cell Mitophagy and Inflammation

Research indicates that inflammation is a key pathological process in the onset and progression of CHD. Under stress conditions, endothelial cells' response to inflammatory cytokines leads to the activation of related signaling pathways, resulting in the release of pro-inflammatory mediators such as TNF-α, IL-1β, and IL-6 [157]. In addition, endothelial cells also increase the expression of adhesion molecules (such as vascular cell adhesion molecule 1 (VCAM-1) and intercellular adhesion molecule 1 (ICAM-1)), which promote the migration of leukocytes (especially monocytes and T cells) to the vascular wall [158]. This migration accelerates subendothelial lipid accumulation, transformation of monocytes to macrophages, and foam cell formation, thereby advancing plaque formation and development [149]. Recent studies reveal that mitophagy in endothelial cells plays a protective role in modulating inflammatory responses, acting against CHD [160]. For example, Yimai Keli activates endothelial cell mitophagy through the PINK1-Mfn2-Parkin pathway, regulating inflammation and vasoconstrictors, thereby alleviating the progression of CHD [161]. Zhou and colleagues observed that in the inflammatory cell infiltration in cardiac microvascular endothelial cells caused by ischemia-reperfusion, melatonin reduces inflammatory cell infiltration by activating mitophagy, thus protecting endothelial cells [155]. PM2.5 is considered a risk factor for CHD [163]. Research by Lai et al. shows that under high glucose conditions, PM2.5 exacerbates damage to endothelial cells, leading to increased levels of inflammatory markers, such as ICAM-1, VCAM-1, and IL-6 [164]. These changes are associated with increased expression of JNK and p38 proteins in the MAPK pathway [164]. However, the elevated expression of LC3B-II and BNIP3 suggests enhanced mitophagy activity, which might be a response to the dual stimuli of high glucose and PM2.5. Ultimately, studies indicate that the use of vitamin D can mitigate damage and inflammatory responses in endothelial cells [164]. Furthermore, research by Li et al. on the effects of microgravity on endothelial cells showed that under microgravity, the NLRP3 inflammasome leads to excessive endothelial cell permeability and migration by releasing IL-1β, while PINK1-dependent mitophagy reduces such inflammatory injury [165]. These studies demonstrate that the activation of mitophagy in endothelial cells can reduce the level of endothelial inflammation, protecting the function and homeostasis of endothelial cells.

### 3.1.3 Endothelial Cell Mitophagy and Other Pathological Changes

In addition to the aforementioned aspects, multiple studies have revealed that mitophagy modulates the function of endothelial cells via various mechanisms, effectively combating CHD. For example, the work of Ma et al. indicates that exercise can enhance FUNDC1-dependent mitophagy in endothelial cells, which in turn delays the aging process of coronary artery endothelium [26]. Similarly, Li et al. also found that a reduction in endothelial cell mitophagy levels leads to endothelial cell aging, which may be a potential mechanism for endothelial aging caused by IDH2 deficiency [154]. Areas of low blood flow shear stress in the vasculature, such as at arterial bifurcations and bends, are more prone to atherosclerosis [166]. The study by Liu et al. confirmed that low shear stress significantly suppresses mitophagy in endothelial cells, thus exacerbating mitochondrial dynamics and providing scientific evidence for the relationship between low blood flow shear stress and atherosclerosis [167].

In summary, these studies emphasize the key role of mitophagy within endothelial cells in preventing and treating CHD. They not only shed light on the mechanisms of CHD formation and progression but also offer valuable insights for the development of therapeutic strategies, pointing towards future research directions.

### 3.2 Ischemic Stroke

Ischemic stroke is an acute cerebrovascular disease, often caused by blood vessel occlusion leading to cerebral tissue ischemia, ultimately resulting in brain cell damage or death [168]. Ischemic strokes account for about 87% of all types of strokes, and with their significant incidence, mortality, and disability rates, they pose a major global health challenge [169, 170]. Reperfusion therapies such as thrombolysis and thrombectomy are the main treatments for ischemic stroke [171]. While greatly reducing mortality, damage caused by ischemia-reperfusion remains a key factor threatening the survival of brain cells [172]. One of the hallmark pathologies of ischemic stroke is the disruption of the blood-brain barrier (BBB), which significantly exacerbates brain injury and subsequent neurological dysfunction [173]. Brain microvascular endothelial cells contribute to the structure and maintenance of the BBB by forming tight junctions, regulating transmembrane transport proteins, and secreting regulatory factors [174]. Recent studies have revealed the critical role of mitophagy in protecting endothelial cells during ischemic stroke, highlighting the regulation of mitophagy in endothelial cells as a key therapeutic strategy against ischemic stroke (IS).

During the ischemic period and I/R (ischemia/reperfusion) injury, damage to brain vascular endothelial cells, impairment of the BBB, mitochondrial dysfunction, and neuronal apoptosis have been observed. Experiments on rat middle cerebral artery occlusion and reperfusion show excessive mitochondrial fission in brain tissue, neuronal apoptosis, and increased oxidative stress [175]. Guo et al., using a rat cerebral ischemia-reperfusion model, found that mitochondrial morphology and function were disrupted [176]. Similar results were demonstrated in in vitro experiments on rat primary cortical neurons subjected to OGD/R (oxygen-glucose deprivation/reperfusion) [175]. In mouse models of middle cerebral artery occlusion for ischemic stroke, death of brain vascular endothelial cells, disruption of tight junction integrity, and damage to the BBB were observed [177]. Furthermore, Wei et al. noted activation of mitophagy and dysfunction in angiogenesis resulting from injury to brain vascular endothelial cells in a rat model of ischemic reperfusion [178].

Further research suggests that during the ischemic reperfusion process, mitophagy in endothelial cells is regulated by multiple mechanisms. The study by Cai et al. found that during brain ischemia-reperfusion, tissue-type plasminogen activator (tPA) can enhance FUNDC1-dependent mitophagy via AMPK phosphorylation, thereby protecting neurons [179]. Additionally, under hypoxic conditions, PINK1/Parkin-dependent mitophagy in endothelial cells is also activated, which helps to reduce ROS production and NLRP3 expression [180]. However, the role of mitophagy during ischemic reperfusion is still controversial. During brain ischemia-reperfusion, LC3B (a marker protein of mitophagy) is primarily expressed in neurons, with lower levels in astrocytes and vascular endothelial cells, as similar conclusions were drawn from in vitro experiments with brain microvascular endothelial cells [181]. These studies suggest that mitophagy in endothelial cells may have both protective and harmful roles during ischemic reperfusion injury, with complex regulatory mechanisms that can be influenced by multiple factors such as the degree of ischemia, reperfusion timing, and inflammatory responses. Future research is needed to further investigate its specific mechanisms of action and find effective regulatory means to mitigate the adverse effects of ischemic reperfusion injury.

Certain compounds can regulate mitophagy in brain vascular endothelial cells when damaged, thereby protecting brain cells. For instance, the active fraction of Polyrhachis vicina (Roger) (AFPR) is a natural compound that can reduce cortical neuron apoptosis and infarct size by targeting the regulation of SIRT3 and activating PINK1/Parkin-mediated brain microvascular endothelial mitophagy, as well as promoting angiogenesis [178]. Ferulic acid (FA) mitigates brain microvascular endothelial cell damage induced by oxygen-glucose deprivation (OGD) via punctate mitochondrial-dependent autophagy, exerting a neuroprotective effect [182]. Tissue-type plasminogen activator (tPA), a commonly used thrombolytic agent in clinical settings, protects neurons in brain microvascular endothelial cells treated with OGD by increasing AMPK phosphorylation levels, which then regulate FUNDC1-mediated mitophagy [179]. Brain-derived neurotrophic factor (BDNF) can activate HIF-1α/BNIP3-mediated mitophagy, protecting endothelial cells from oxidative stress and apoptosis in high glucose-induced brain microvascular endothelial cell injury [182]. Methylglyoxal (MG), which is elevated in the blood of diabetic patients, increases mitochondrial ROS production and suppresses the Akt/HIF-1α pathway in brain endothelial cells treated with MG, while enhancing Parkin-mediated mitophagy [183]. N-acetylcysteine can significantly reverse mitochondrial damage and autophagy, protecting endothelial cells from MG damage [1783]. These findings open new avenues for exploring candidate compounds that regulate brain vascular endothelial cell mitophagy and offer potential directions for new drug development.

In summary, although a substantial amount of research indicates the protective effects of mitophagy on ischemic reperfusion (IR) injury, mitophagy in endothelial cells exhibits dual characteristics of a double-edged sword. Therefore, future studies need to delve deeper to reveal more detailed mechanisms of action.

### 3.3 Diabetic Vascular Complications

Diabetic vascular complications are caused by long-term high blood sugar, which leads to vascular damage affecting multiple organs including the heart, brain, and kidneys, resulting in a series of serious health problems [184]. Endothelial cells, located in the inner layer of blood vessels, are directly exposed to the high blood sugar environment in the bloodstream. This continuous stimulation significantly affects the function and structure of endothelial cells, thereby accelerating the development of vascular complications [185]. Impaired endothelial cell function due to prolonged high blood sugar is a key factor in the early development of diabetic vascular complications [186]. This includes a decrease in endothelium-dependent vascular dilation caused by reduced generation of nitric oxide (NO), increased expression of pro-inflammatory cell adhesion molecules, and changes in vascular permeability [187]. Additionally, high blood sugar directly damages endothelial cells, increases oxidative stress, and further promotes vascular injury and remodeling [188]. Therefore, regulating the function of endothelial cells is a key strategy for preventing and treating diabetic vascular complications.

Numerous studies have shown that mitophagy plays a significant role in endothelial cell damage in diabetic vascular lesions. Under high-sugar conditions, endothelial cells in cardiac microvessels undergo ferroptosis, and Pink1/Parkin-dependent mitophagy is observed to be inhibited [189]. Similarly, under conditions of high sugar stress and ischemia-reperfusion (IR) injury, the mitochondrial autophagic function of rat pulmonary microvascular endothelial cells is impaired, accompanied by increased oxidative stress, inflammatory response, and apoptosis [190]. Research on diabetic cardiomyopathy has also shown that a high-sugar environment inhibits PINK1-Parkin-dependent mitophagy in endothelial cells [191]. Likewise, in glomerular endothelial cells, high sugar stimulation leads to impaired mitophagy [192]. Furthermore, under conditions of high blood sugar and hyperlipidemia, rat aortic endothelial cells exhibit impaired mitophagy after exposure to high sugar and palmitic acid ester treatment [193]. These findings suggest that under various environmental conditions, high sugar status can lead to damaged mitophagy in endothelial cells, with accumulated damaged mitochondria exacerbating endothelial cell damage. However, there are also studies indicating that a high sugar environment can induce mitophagy. For example, in primary endothelial cells exposed to high concentrations of glucose, there is a significant increase in mitochondrial quantity, decreased mitochondrial network interconnectivity, and increased expression levels of Pink1 and Parkin genes associated with mitophagy [194]. Meanwhile, in endothelial cells co-treated with high sugar and particulate matter (PM), levels of reactive oxygen species (ROS) in mitochondria increase, promoting activation of mitochondrial division and autophagy [160]. This activation may be a self-protective mechanism initiated by endothelial cells to eliminate damaged mitochondria.

Mitophagy in endothelial cells is a key pathological change in diabetic vascular complications [195]. Therefore, regulating mitophagy in endothelial cells can serve as an effective strategy for treating these complications. Several molecules have shown potential in regulating mitophagy. For example, Scutellarin, the main active ingredient of Erigeron breviscapus, can enhance PINK1/Parkin-dependent mitophagy in high-glucose-induced human umbilical vein endothelial cells, reducing ROS levels and the expression of P62, Cyt.c, and Cleaved caspase-3, thereby protecting cells from damage in a high-glucose environment [196]. Additionally, mesenchymal stem cells (MSCs) have also demonstrated a protective effect on high-glucose-damaged endothelial cells, possibly through the activation of Pink1/Parkin-dependent mitophagy [197]. Furthermore, specific deletion of Fgf13 in renal tubular endothelial cells improves mitochondrial homeostasis and endothelial barrier integrity in type 2 diabetic nephropathy (T2DN), as the absence of Fgf13 enhances the action of Parkin, promotes mitophagy, and reduces cell apoptosis, indicating its potential value as a therapeutic target for T2DN [192]. Other compounds such as l-carnitine [191], BDNF [198], and adiponectin [190] also activate mitophagy in endothelial cells through different mechanisms, exerting protective effects. Liraglutide, a glucagon-like peptide-1 (GLP-1) receptor agonist, has been shown in Zhang et al.'s study to reduce PINK1 expression and Parkin accumulation in human umbilical vein endothelial cells treated with high glucose, inhibiting excessive mitophagy, protecting the endothelial barrier, and alleviating the impact of high glucose on endothelial function [199]. Nano-curcumin reduces excessive mitophagy in endothelial cells treated with high glucose and particulate matter, improving ROS and inflammation levels [1560]. Salvianolic acid B reduces excessive mitophagy in endothelial cells induced by high glucose and oxidized low-density lipoprotein by downregulating ROCK1 [200]. Additionally, some natural herbal medicines can also be used to regulate endothelial cells. For instance, endothelial cell senescence induced by HG/PA leads to endothelial dysfunction, which in turn causes diabetic cardiovascular complications. Ginseng-Sanqi-Chuanxiong stimulates mitochondrial autophagy through the AMPK pathway, thereby preventing endothelial senescence and the production of mtROS induced by HG/PA [195]. These studies provide a range of candidate compounds and molecular targets for the treatment of diabetic vascular complications, while also indicating directions for new drug development. This not only aids in designing targeted therapeutic strategies but also promotes a deeper understanding of the mechanisms of diabetic vascular complication treatment, opening up new possibilities for future drug development.

Although regulating mitophagy has shown therapeutic potential in various diseases, the mechanisms by which mitophagy operates in different cardiovascular diseases exhibit significant differences. These differences reflect the impact of disease-specific contexts on mitophagy. For instance, in atherosclerosis, impaired mitophagy in endothelial cells and vascular smooth muscle cells is a key factor in disease progression [114]. Atherosclerosis formation is closely related to the accumulation of LDL and oxidative stress. Studies have shown that oxidized LDL can inhibit mitophagy, leading to mitochondrial dysfunction in endothelial cells and vascular smooth muscle cells, thereby promoting inflammation and plaque formation. In this context, dysregulation of the PINK1/Parkin pathway can result in mitochondrial depolarization and cell apoptosis, accelerating the progression of atherosclerosis [114]. In the case of stroke, abnormalities in mitophagy are also closely related to the severity of brain tissue damage. During ischemia-reperfusion, inhibition of mitophagy leads to excessive mitochondrial damage and cell death. Research indicates that mitophagy protects neurons from excessive oxidative stress and cell death by removing damaged mitochondria [201]. Diabetic vascular complications are closely associated with hyperglycemia and related oxidative stress and inflammation [186]. In diabetes, dysregulated mitophagy is linked to endothelial dysfunction and increased vascular inflammation. In hyperglycemic conditions, advanced glycation end products (AGEs) can interfere with mitophagy, resulting in mitochondrial dysfunction and exacerbating vascular damage [202]. Moreover, the impaired mitophagy function in diabetic patients makes it difficult to clear damaged mitochondria, further causing endothelial cell dysfunction and progression of vascular complications. These differences highlight the mechanistic specificity of mitophagy in different cardiovascular disease contexts, suggesting that future therapeutic strategies should focus on individualized interventions targeting autophagy regulation in specific disease settings.

## 4. Potential therapeutic strategies for mitophagy

Through an analysis of the current research findings, we propose potential therapeutic strategies targeting mitochondrial autophagy, including approved pharmaceuticals, emerging small molecule compounds under development, lifestyle modifications, and gene editing technologies.

### 4.1 Approved Pharmaceuticals

Rapamycin inhibits the mTOR pathway, activating autophagy, including mitophagy, which helps clear damaged mitochondria and improve cell function. Clinical trials have shown Rapamycin's efficacy in cardiovascular diseases, such as reducing cardiac remodeling and heart failure incidence in myocardial infarction patients [203], decreasing arterial plaque formation and cardiovascular event risk in atherosclerosis patients [204]. These findings suggest Rapamycin effectively improves cardiovascular outcomes by activating mitophagy.

Metformin, a common diabetes drug, promotes mitophagy by activating AMPK, which helps maintain cellular energy balance and upregulates PINK1/Parkin pathways to remove damaged mitochondria. Research shows Metformin significantly increases mitophagy in endothelial cells, reducing oxidative stress and apoptosis [205]. Clinical studies demonstrate Metformin's cardiovascular benefits, including a lower incidence of cardiovascular events in type 2 diabetes patients [206] and improved heart function in coronary artery disease patients [207], indicating its potential in improving cardiovascular outcomes through mitophagy regulation.

Empagliflozin, an SGLT2 inhibitor, has been found to activate mitophagy by stimulating AMPK. It reduces oxidative stress and apoptosis in cardiomyocytes [208]. Clinical trials show Empagliflozin's cardiovascular protective effects, including reduced hospitalization rates and cardiovascular mortality in heart failure patients [209], and decreased atherosclerotic plaque formation and cardiovascular event risk in diabetes patients [210]. These results highlight Empagliflozin as a promising strategy for cardiovascular disease treatment through mitophagy activation.

Melatonin, a natural hormone, has been shown to activate mitophagy by regulating the SIRT1/PGC-1α pathway, reducing oxidative stress and apoptosis in cardiomyocytes [211]. Clinical trials reveal Melatonin's cardiovascular benefits, including improved heart function and reduced heart failure incidence in myocardial infarction patients [212], and lower blood pressure and cardiovascular event risk in hypertension patients [213]. This suggests Melatonin as a potential treatment for cardiovascular diseases through its role in mitophagy activation.

### 4.2 Emerging small molecule compounds under development

Urolithin A, a natural compound found in certain fruits, has shown potential in regulating mitochondrial autophagy. It activates mitochondrial autophagy, reducing oxidative stress in myocardial ischemia-reperfusion injury and protecting the cardiovascular system [150]. Urolithin A enhances mitochondrial biogenesis and function by upregulating Sirtuin 3 and PGC-1α, improving endothelial cell survival and proliferation [150]. In a clinical trial in middle-aged and older adults, Urolithin A showed good mitochondrial protection and anti-inflammatory effects [214]. Resveratrol promotes mitochondrial autophagy through AMPK and SIRT1 pathways, reducing oxidative stress and inflammation in cardiomyocytes, and improving cardiovascular function [215]. It also regulates the PINK1/Parkin pathway, enhancing mitochondrial quality control and preventing cardiomyocyte apoptosis [123]. Ferulic Acid, a phenolic acid present in plants, has antioxidant and anti-inflammatory properties. It promotes mitochondrial autophagy via the Nrf2/ARE pathway, reducing oxidative stress and inflammation in endothelial cells [216], and maintains mitochondrial function and structure by modulating fusion and fission processes [217]. Scutellarin, a flavonoid extracted from the traditional herb Scutellaria, activates PINK1/Parkin-mediated mitochondrial autophagy, mitigating high-glucose-induced endothelial cell damage and protecting the cardiovascular system. It also inhibits mitochondrial apoptosis, enhancing endothelial cell survival [196]. These findings support these small molecule compounds as a viable cardiovascular treatment strategy.

### 4.3 Lifestyle modifications

Lifestyle factors, such as exercise, diet, and smoking habits, significantly impact mitophagy. A sedentary lifestyle and a high-fat diet can lead to mitochondrial dysfunction in endothelial cells, increasing oxidative stress and inflammation, thus disrupting mitophagy [218]. Lifestyle improvement as a non-pharmacological intervention is widely recognized to improve cardiovascular health and reduce overall mortality from cardiovascular disease [219]. For instance, aerobic exercise enhances cardiac function in CVD rat models by increasing M2 acetylcholine receptor (M2AChR) expression [220]. Additionally, it modulates mitochondrial protein homeostasis, slowing Alzheimer’s disease progression [221]. Aerobic exercise significantly improves cardiac function and mitochondrial quality in CVD prevention and treatment [222]. Systematic reviews and meta-analyses further confirm that aerobic exercise enhances mitochondrial oxidative capacity in CVD patients [222]. Overall, aerobic exercise plays a crucial role in CVD treatment by activating mitochondrial autophagy through various mechanisms.

### 4.4 Gene editing technologies

Genetic factors play a significant role in mitophagy within endothelial cells. For example, certain genetic variations can affect the expression and function of mitophagy-related genes such as PINK1 and Parkin [83]. Studies have found that mutations in these genes may lead to mitophagy dysfunction, increasing the risk of cardiovascular diseases [223]. Gene editing technologies, particularly CRISPR/Cas9, hold great promise for treating mitochondrial autophagy gene defects. Recent advances in CRISPR/Cas9 have enabled precise repair or replacement of defective genes. For example, knockout of the Slc25a46 gene in mice models mimics aging-related phenotypes, such as impaired motor function and mitochondrial dysfunction, providing a platform for studying aging-related pathology and developing mitochondrial quality-improvement strategies [224]. Additionally, gene editing techniques are being explored for mitochondrial genome editing. By correcting or replacing mtDNA mutations, researchers can improve mitochondrial function25, as demonstrated by CRISPR/Cas9's specific editing of point mutations in the GBA gene, without affecting its pseudogene GBAP1 [226]. These advancements offer new approaches to addressing gene editing complexities caused by pseudogenes.

## 5. Problems and disputes

Although endothelial cell mitophagy is crucial for cardiovascular diseases, its role in these conditions is complex and controversial. Firstly, excessive mitophagy might lead to endothelial cell dysfunction. Some studies have found that over-clearing mitochondria can cause energy deficiencies, affecting cell survival and function, thereby exacerbating the progression of cardiovascular diseases [227]. On the other hand, some studies suggest that the process of mitophagy might release endogenous danger signals (such as mitochondrial DNA), which can trigger immune and inflammatory responses [108]. In such cases, mitophagy could exacerbate endothelial cell inflammation, further promoting the development of cardiovascular diseases. Additionally, different types of cardiovascular diseases (such as atherosclerosis, hypertension, and heart failure) may respond differently to mitophagy [115]. Some studies indicate that under specific pathological conditions, mitophagy may have a protective role, while under other conditions, it might have a disease-promoting effect [228]. This diversity and complexity in mechanisms increase the difficulty of research. Therefore, further studies are needed to clarify the dual roles of mitophagy in cardiovascular diseases and explore how to effectively regulate this process to achieve optimal therapeutic outcomes.

Second, a range of ethical, regulatory and safety issues must be considered when discussing mitochondrial autophagy as a potential strategy for the treatment of cardiovascular disease. These issues not only involve the use of drugs and small molecule compounds but also include lifestyle adjustments and the application of gene editing technologies. Firstly, existing drugs such as rapamycin, metformin, and empagliflozin have shown potential in activating mitophagy, but their long-term use may bring side effects. Therefore, in clinical applications, these drugs' side effects must be strictly monitored, and individualized adjustments should be made according to the specific conditions of the patients. Second, small molecule compounds under development, such as urolithin A, resveratrol, and ferulic acid, show promise in activating mitophagy, but their safety and efficacy still need further validation. For instance, urolithin A has demonstrated significant mitochondrial protective effects in animal models, but its long-term safety in humans remains unclear. Resveratrol, while having antioxidant and anti-inflammatory properties, has low bioavailability, which necessitates the development of more effective delivery systems [229]. Ferulic acid shows potential in cardiovascular protection, but its specific mechanisms and long-term safety require further research. Hence, before applying these small molecule compounds in clinical practice, comprehensive clinical trials must be conducted to verify their safety and efficacy. Lifestyle adjustments, such as aerobic exercise and dietary control, also show positive effects in activating mitophagy, but their implementation faces challenges. Although aerobic exercise has been proven to improve cardiovascular health, its effects vary due to individual differences, and it may not be suitable for all patients. Finally, gene editing technology shows great potential in treating diseases related to mitophagy, but its ethical and safety issues cannot be ignored. Gene editing technologies, such as CRISPR-Cas9, can precisely repair gene defects, but their off-target effects and long-term safety require further research [230]. Additionally, the application of gene editing technologies involves ethical issues, such as the fairness and accessibility of gene editing [231]. Therefore, before applying gene editing technology in clinical practice, thorough ethical reviews and safety evaluations must be conducted.

Finally, in studying endothelial cell mitophagy and aging-related cardiovascular diseases, the advantages and limitations of various methodologies need to be carefully weighed. Firstly, accurate selection of experimental models is crucial for studying endothelial cell mitophagy. Commonly used models include in vitro cultured endothelial cells, animal models, and clinical samples. In vitro cultured cell models facilitate manipulation and observation of the details of mitophagy, but may not fully simulate the complex in vivo environment. Animal models, such as mice and rats, provide a closer approximation to physiological conditions, but their results may be affected by species differences. Clinical samples can provide direct pathological information but are difficult to obtain and usually limited in sample size. Secondly, appropriate labeling and detection methods are necessary to monitor mitophagy in experimental design. Common methods include immunofluorescence staining, electron microscopy, flow cytometry, and fluorescent probes. Immunofluorescence staining can locate and quantify specific autophagy markers, such as LC3 and P62, but may be affected by non-specific background [232]. Electron microscopy provides detailed ultrastructural information and is considered the "gold standard" for observing mitochondria and autophagosomes, but is complex and time-consuming [233]. Flow cytometry and fluorescent probes can be used for high-throughput analysis but may require optimization to ensure specificity and sensitivity [234, 235]. Additionally, a key issue in experimental design is how to validate the reproducibility and reliability of experimental results. This typically requires conducting multiple independent experiments and verifying results under different conditions. Furthermore, establishing control and experimental groups is crucial to eliminate interference from other factors. Choosing appropriate controls and standardizing experimental procedures is essential to ensure the reliability of research data. By carefully selecting and combining these methods, a more comprehensive understanding of the role of endothelial cells in aging and cardiovascular diseases can be achieved, and progress in the relevant field of research can be advanced.

## 6. Future Directions

Despite the variety of targets, compounds, and technologies available for the treatment of cardiovascular diseases, we are still far from curing them. Therefore, future progress can be made by employing more scientifically advanced methods to facilitate the translation of these research findings into clinical practice.

### 6.1 Phenotype High-Throughput Screening for Treating Endothelial Cell Mitochondrial Dysfunction

Phenotype high-throughput screening (HTS) technology is a powerful tool that allows for the rapid identification of potential therapeutic drugs and targets. In recent years, HTS technology has made significant advances in addressing endothelial cell mitochondrial dysfunction. Firstly, HTS technology can screen large compound libraries to discover drugs with therapeutic potential. Secondly, HTS can be used to identify gene targets, revealing the molecular mechanisms underlying endothelial cell mitochondrial dysfunction. Additionally, HTS can be combined with other omics technologies, such as genomics, proteomics, and metabolomics, to comprehensively analyze the complex network of endothelial cell mitochondrial dysfunction. Endothelial cell mitochondrial dysfunction plays a crucial role in various cardiovascular diseases. Through high-throughput screening, researchers can rapidly identify potential therapeutic drugs. Perea-Gil et al. developed a phenotypic screening platform using patient-specific induced pluripotent stem cell (iPSC)-derived cardiomyocytes and discovered two small molecule kinase inhibitors (SMKIs) that significantly improved contraction and mitochondrial respiration functions in dilated cardiomyopathy (DCM) cells with different genetic mutations [236]. This approach not only revealed new therapeutic targets but also provided new ideas for personalized treatment. Therefore, future research could further optimize HTS technology, integrate other omics techniques, and delve deeper into the molecular mechanisms of endothelial cell mitochondrial dysfunction to develop more effective therapeutic strategies.

### 6.2 Omics Approaches Using ECFCs for Drug and Biomarker Discovery

In recent years, the application of endothelial colony-forming cells (ECFCs) in drug and biomarker discovery has garnered widespread attention. ECFCs, with their high proliferation ability and expression of endothelial-specific markers, are ideal models for studying angiogenesis and endothelial dysfunction. Omics approaches, including genomics, transcriptomics, proteomics, and metabolomics, provide comprehensive biological information that helps uncover disease mechanisms and potential therapeutic targets [237]. By performing omics analyses on ECFCs, key genes, proteins, and metabolites associated with diseases can be identified, providing new insights for drug development and biomarker discovery [238]. Gerasimov et al. used high-throughput sequencing technology to investigate mitochondrial RNA editing and transcriptomic variations under different stress conditions in various Trypanosoma strains, revealing the critical role of RNA editing in regulating mitochondrial gene expression and metabolic capacity [238]. Such omics methods offer new perspectives on understanding the role of ECFCs in disease mechanisms.

### 6.3 Targeted Gene Editing Technologies

Targeted gene editing technologies have made significant advances in recent years, particularly with the application of the CRISPR-Cas9 system, which has made genome editing more efficient and precise. The CRISPR-Cas9 system, derived from bacterial adaptive immune systems, uses guide RNA (gRNA) to recognize specific DNA sequences and employs the Cas9 nuclease to make targeted genome modifications [239]. Silva-Pinheiro et al. successfully achieved in vivo mitochondrial DNA editing in mouse hearts by delivering a double-stranded DNA deaminase (DddA)-derived cytosine base editor (DdCBE) using adeno-associated virus (AAV) vectors, providing crucial experimental evidence for future gene therapy [240]. Furthermore, targeted gene editing has enabled the construction of more optimized disease models in basic research, providing robust technical support for foundational studies.

## 7. Conclusion

Aging is an inevitable risk factor for human diseases [10]. Endothelial cells are crucial components of the cardiovascular system, and their function gradually deteriorates with age [25]. Given the relatively low mitochondrial content in endothelial cells, controlling mitochondrial quality is particularly crucial [20]. Mitophagy, as a core mechanism of mitochondrial control, timely clears damaged and aging mitochondria, maintaining mitochondrial homeostasis [20]. However, with advancing age, mitophagy function may be impaired, leading to further endothelial dysfunction, becoming a potential pathogenic mechanism for various cardiovascular diseases [26, 118, 121, 122]. Therefore, regulating mitophagy in endothelial cells is an important strategy for addressing age-related diseases. Future studies should focus on how to restore mitochondrial autophagy function of endothelial cells through drugs or gene editing techniques, with a view to improving cardiovascular health and delaying the aging process.
